# Fossil bacula of five species of Borophaginae (Family: Canidae): Implications for their reproductive biology

**DOI:** 10.1371/journal.pone.0280327

**Published:** 2023-01-17

**Authors:** Daniel Varajão de Latorre

**Affiliations:** 1 Department of Integrative Biology, University of California, Berkeley, Berkeley, California, United States of America; 2 University of California Museum of Paleontology, Berkeley, California, United States of America; Universita degli Studi della Basilicata, ITALY

## Abstract

The baculum of mammals offers the opportunity to study the reproductive biology of extinct species given that it is a fossilizable part of the male genitalia and that its size and shape correlate with several aspects of the reproductive biology of extant mammals. Fossil bacula, however, are rare. Currently, bacula have been described from only two extinct species of canids, one from the subfamily Caninae and the other from the extinct subfamily Hesperocyoninae. Here, I describe the bacula of five extinct species of Borophaginae, each of which was found with other skeletal elements that have enabled identification to the species level. Two specimens (*Aelurodon ferox* and *Aelurodon stirtoni*) are largely complete, while the baculum from *Carpocyon compressus* is complete but still embedded in matrix that obscures some of its features. The bacula of *Paratomarctus euthos* and *Desmocyon thomsoni* are incomplete, but they provide useful information nonetheless. These borophagine bacula are similar to extant canines in being robust, having a urethral groove, and a simple distal end. These features suggest that the Borophaginae had long-lasting copulation and possibly spontaneous ovulation, similar to the extant canines. However, unlike the straight baculum of extant canines, borophagine bacula are ventrally curved (arched), which is also observed in the hesperocyonine baculum. The implication of this curvature for the reproductive biology of these animals remains unknown.

## Introduction

Studying the reproductive biology of extinct animal groups is challenging. While neontologists can observe directly the morphology, behavior, endocrine responses, and life cycles of their study organisms, it is extremely rare for paleontologists to find direct evidence of reproductive biology [*e.g.*, a fossilized pair of mating insects 1]. Instead, paleontologists must rely largely on morphological traits that fossilize, and on the fidelity of correlations between these traits and other aspects of reproduction seen among extant species. As a result, we have much less information regarding the reproductive biology of extinct species than for living species. Consistent with this, sexual selection and mating represent less than 5% of all the entries in the compendium of behaviors inferred from fossils [Hsieh and Plotnick, [2], tabulated Boucot and Poinar 1’s, [3], compendium]. Nonetheless, when aspects of reproductive biology can be inferred, they can provide important insights into observed changes in biodiversity. For example, Bush *et al.* [4] showed that marine animals with direct fertilization drive the diversification of marine fauna during the Cretaceous-Cenozoic while groups that spawn in the water column have a relatively constant diversity over the same period.

In this context, the penis bone of mammals (baculum, *os penis* or *os priapi*) provides an opportunity for drawing inferences regarding the reproductive biology of extinct species [*e.g.*, 5]. The baculum is present in 7 mammalian orders, within which it has been gained and lost multiple times [6]. Interestingly, females of some species have *ossa genitalia* homologous to the baculum, the baubellum or *os clitoridis*, but it is normally smaller than the male counterpart [*e.g.*, 7] and is largely understudied, to such an extent that it has not been associated with any aspect of animals’ reproductive biology. The baculum can inform our understanding of the reproductive biology of extinct species because it is a part of the male genitalia with potential for fossilization and because its morphology is known to correlate with several aspects of the reproductive biology of extant mammals. For example, in carnivorans induced ovulation tends to occur in species that have bacula with a more complex distal tip [8]. Moreover, more robust bacula are associated with prolonged copulation [9], and longer bacula are associated with larger testis [8, 10], which is indicative of stronger sperm competition [11].

A good fossil record for carnivoran bacula would allow us to explore how the reproductive biology of this mammalian clade has changed through time. However, very few extinct species have had bacula described [but see 5, 12–17, for the description of the baculum in eight species]. To date, bacula have only been described from two extinct canid species: *gregarius* in the extinct subfamily Hesperocyoninae [16], and the dire wolf, *Aenocyon dirus* [17, 18], in the still extant subfamily Caninae. Here, I add to our knowledge of canid bacula by describing the bacula of five species of Borophaginae, the other extinct subfamily of Canidae. All specimens are from North America and were found associated with sufficient other skeletal elements that allowed identification to the species level by Wang et al. [19]. Without the association with other skeletal material, species level identification would not be possible, as fossil bacula are rare and lack diagnostic traits. Although there is always a chance for misidentification of fossils identified from associated elements, I consider that the chances are low for the specimens described here, given previous work on these specimens [19] and considering that none of them are from fossil rich localities or bone beds. In fact, most of the specimens are the only ones from the localities in which they were found. These are the first descriptions of bacula that can be reliably attributed to Borophaginae.

The subfamily Borophaginae has a rich fossil record, spanning over 30 Ma, from the Orellan (base around 34 Ma, early Oligocene) to the Blancan (top around 1.8 Ma, early Pleistocene) with at least 66 recognized species [19]. They were the most diverse group of carnivores in the middle Miocene in North America [20], and by that time had radiated from a small hypocarnivorous ancestor to exhibit a diverse range of body sizes and diets [21]. Functional morphological studies have led to opposing conclusions regarding whether they formed groups to hunt or not [22, 23]. Currently, very little is known about their reproductive biology—the bacula described here offers new insights into this aspect of their biology.

## Bacula of extant canids

**Fig 1:**
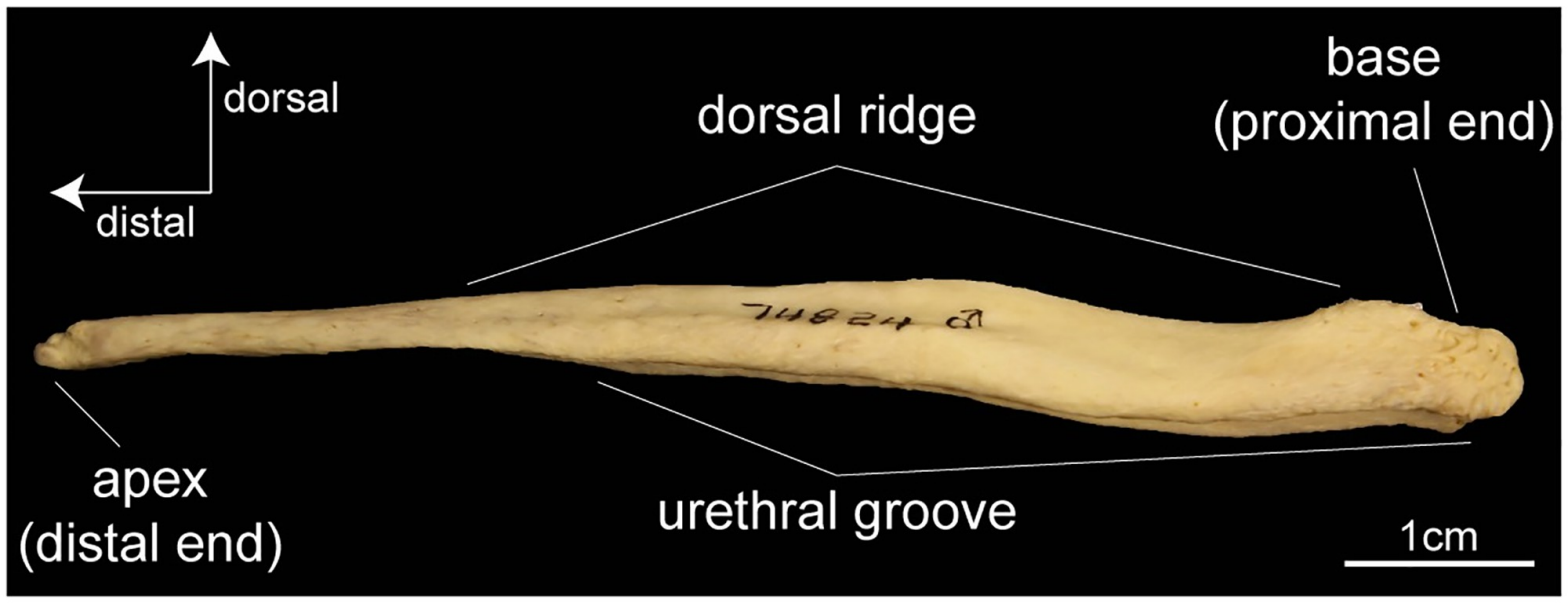
Canine baculum. Left lateral view of the baculum of coyote (*Canis latrans*) with visible urethral groove on the ventral side, a dorsal ridge, and soft tissue attachment scars on the base. (Specimen 74824 from the Museum of Vertebrate Zoology, Berkeley, CA, USA).

I use the bacula of the extant canids [all from subfamily Caninae, 24–26] as a reference for the anatomy of Borophaginae bacula. Most extant canines have a fairly straight baculum in lateral profile, although it may curve slightly ventrally (concave down) or dorsally (concave up). The baculum is a distal continuation of the high pressure erectile tissue named corpora cavernosa [27, 28], and the base of the baculum (proximal end) shows marks of soft tissue attachment (scars). On the ventral side there is a deep groove that runs from the proximal end to about three quarters down the length of the shaft (Fig 1). The urethra sits within this groove. The presence of the groove is associated with lateral expansions of the baculum along its ventral margin, which also results in a distinctive proximo-distal dorsal crest. Most individuals have a peak somewhere along the proximal-half of the dorsal crest, but it is absent in some specimens. Further, the bulbus glandis, another high pressure erectile tissue present in extant canids and involved in the copulatory tie of canids, engorges around the proximal three quarters of the baculum, where the urethral groove is present [28]. The distal end (anterior) of the baculum is simple in canids, having either a cylindrical rod-like shape, or being slightly expanded dorso-ventrally. The cartilaginous distal-most portion of the baculum, the apex, can calcify with age [25] but is rarely observed in zoological collections.

## Descriptions of borophagine bacula

**Museum Abbreviations:** AMNH—American Museum of Natural History, New York, NY, US; F:AM—Frick Collection, Department of Vertebrate Paleontology, American Museum of Natural History, New York, NY, USA; USNM PAL—Smithsonian Institution National Museum of Natural History Paleontology collections, Washington, D.C., USA; UNSM—University of Nebraska State Museum, Lincoln, NE, USA.

### Paratomarctos euthos

**Fig 2:**
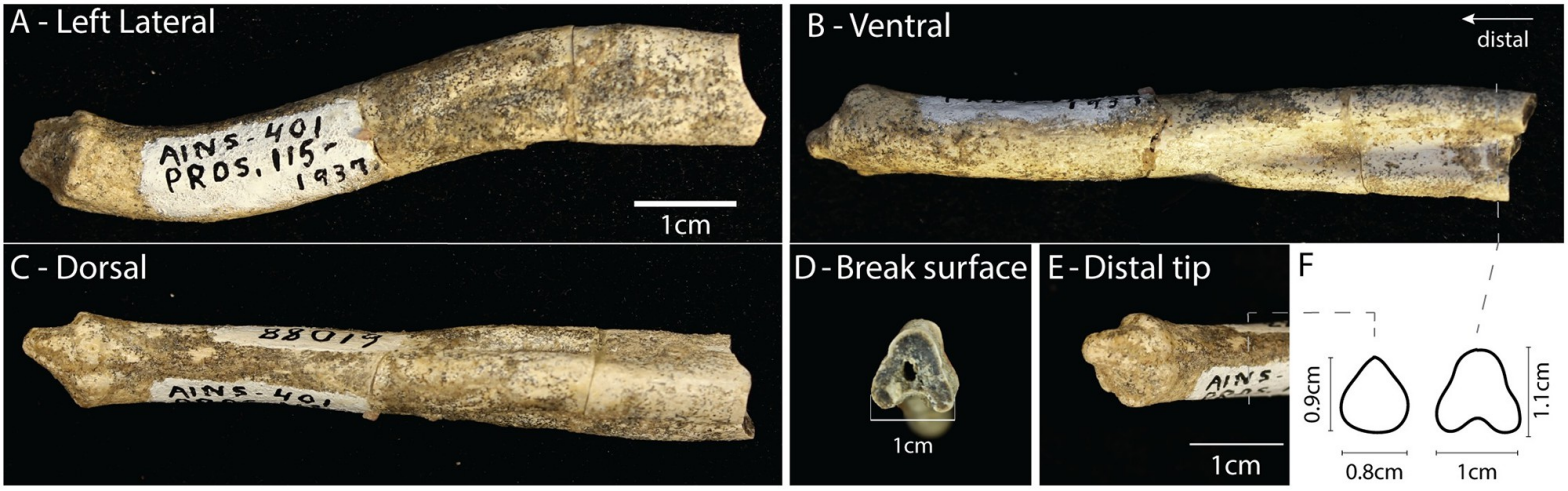
Paratomarctos euthos. The partially preserved baculum of specimen F:AM 61088, exhibiting the complete distal end. A) Left lateral view showing the sigmoidal profile of the bone. B) Ventral view showing the beginning of the ventral groove about half way down the specimen, which then expands proximally. C) Dorsal view showing the lateral expansion where the ventral groove is present and the resulting dorsal crest. A,B and C are at the same scale; anterior at left. D) Slightly oblique view of the broken end. E) Oblique view of distal tip. F) Inferred cross-sections at two points along the specimen with caliper measurements indicated.

*Paratomarctus euthos* was a coyote-sized species [29] that can be considered mesocarnivorous based on its dentition [21], with a tendency towards hypercarnivory [19] and that could take prey larger then themselves [22]. The distal portion of the baculum of *Paratomarctus euthos* (Fig 2) was found associated with fragments of the skull and mandible as well as numerous post-cranial bones [specimen F:AM 61088—19]. This specimen is from the late Clarendonian (13.6-10.3Ma, middle Miocene) from the Merritt Dam Member of the Ash Hollow Formation, Cherry Co., Nebraska. This partial skeleton is the only specimen from its locality in the AMNH collections. The preserved portion of the baculum is 7cm long. Despite the absence of the proximal end, it is clear that the baculum was robust. The ventral side of the proximal portion exhibits a shallow and wide urethral groove, with a corresponding dorsal crest. Distally, the groove becomes gradually shallower and narrower until it disappears about half way along the preserved portion of the baculum. The distal end consists of a small conical projection of rough bone. At the base of this cone there is an almost continuous circumferential ring of bone. The baculum is sigmoidal in lateral profile. There is an overall ventral curvature (concave down) that changes to a dorsal curvature (concave up) at the distal third of the specimen. Given the aspect ratio (length to width) of the specimen it seems likely that the fragment represents at least 50% of the total length of the baculum.

### Desmocyon thomsoni

**Fig 3:**
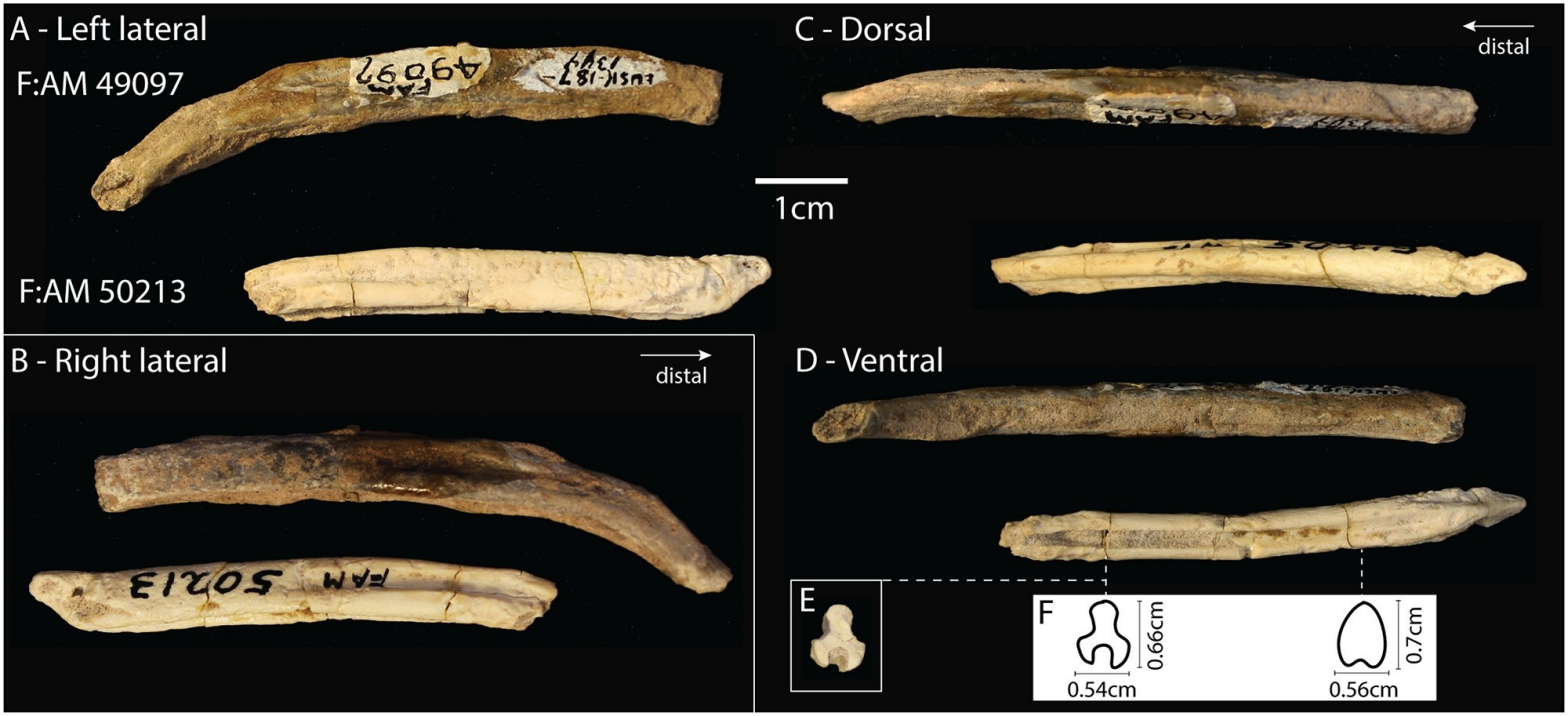
Desmocyon thomsoni. Bacula from specimens F:AM 49097 (upper image in each panel) and F:AM 50213 (lower image in each panel). In each panel the specimens are roughly aligned based on the proximal ends of dorsal crest—it appears that F:AM 49097 is only missing a small portion of its proximal end, while F:AM 50213 is missing a larger portion of its distal end. A) Left lateral view, showing the ventral curvature in F:AM 49097 and soft tissue attachment marks on the proximal end of F:AM 50213. B) Right lateral view. The right side of specimen F:AM 49097 is less abraded giving a better view of the dorsal crest, which is absent from the proximal and distal ends. C) Dorsal view shows the dorsal crest of both specimens, and the triangular distal end of F:AM 50213. D) Ventral view showing the urethral groove in both specimens, although it is filled with matrix in specimen F:AM 49097. The distal surface for this specimen also shows that the specimen broke distally to the distal end of the groove. E) Cross section of the baculum F:AM 50213 where there is a clean break in the specimen. F) Inferred cross sections with caliper measurements indicated.

*Desmocyon thomsoni* was a medium-sized [around 7kg—22] mesocarnivore species that ranged from late Arikareean to early Hemingfordian. Its dental morphology is intermediate between the earlier hypocarnivorous borophagines and the larger hypercarnivorous species common in the later diversification of the subfamily [19, 21, 22]. Two partial bacula of *Desmocyon thomsoni* (Fig 3) are associated with partial skeletons, including partial skulls and mandibles as well as many elements of the post-crania skeletons [19]. Both are from the late Arikareean (30.8—20.43Ma, Oligocene-Miocene) of Wyoming. Specimen F:AM 49097 was found in the Lusk area, Upper Harrison beds in Goshen Co., Wyoming, and it is the only specimen from its locality in the AMNH collections. Specimen F:AM 50213 is also from the Lusk area, Upper Harrison beds, but in Niobrara Co., Wyoming. Only one other specimen (identified as *Promartes*) was found in the same locality of F:AM 50213.

Specimen F:AM 50213 has a complete proximal end where marks of soft tissue attachment are present on the lateral surface. These marks form a 1.5 cm long diagonal line that extends from the dorsal and proximal extremity to the ventral face of the shaft. On the lateral surface, and dorsal to this diagonal line, the bone is rougher but transitions to smoother bone distally. The fragment is mostly straight in lateral profile, except for a slight ventral curvature that starts about one centimeter from the broken end of the specimen. In ventral view there is a deep urethral groove that is narrow and cylindrical. The proximal end of the baculum is laterally compressed, corresponding to where the urethral groove disappears. This differs from most modern canids, in which the urethral groove extends all the way to the proximal end, but is similar to the baculum of the gray fox, *Urocyon cinereoargenteus* [26]. The dorsal surface of the baculum presents a strong proximal-distal crest, which can be seen clearly in the cross section of the broken surface (Fig 3E).

Specimen F:AM 49097 is slightly longer than F:AM 50213 (6.9 cm and 5.63 cm, respectively), but it is more abraded and is missing both the proximal and the distal ends. This specimen preserves more of the distal end than F:AM 50213, revealing a ventral curvature (concave down). The groove is not visible on the either the proximal or distal broken surfaces and is thus restricted to the middle portion of the bone; it is filled with sediment, which obscures the ends of the groove. The dorsal crest is present in the middle portion of the fragment, but not at the proximal and distal ends. Using the proximal end of the crest and lack of the urethral groove on the proximal break surface as reference points to align the two specimens, it is possible to infer that only a small portion of the proximal end of F:AM 49097 is missing. It is not possible to see where the groove ends distally due to sediment and abrasion, but the groove is not present on the distal break surface (as visible in Fig 3D). The specimen starts to taper distally and the dorsal crest is not present along the distal portion. Given these features of the distal portion and given that other canid bacula have fairly simple distal ends, it seems likely that only a small fraction of the distal end of this specimen is missing.

### Aelurodon ferox

*Aelurodon ferox* is among the largest described species of borophagines, with an estimated body mass around 31kg [22]. It is considered a hypercarnivorous species that could take prey larger then themselves and with adaptations for crushing bone [19, 22]. One baculum of *Aelurodon ferox* was found together with a partial skeleton, specimen F:AM 61723, which has a partial mandible and numerous post cranial bones [19]. The specimen is considered Clarendonian in age (13.6-10.3Ma, middle Miocene), from the Pojoaque Member of the Tesuque Formation (Santa Cruz Grant, Santa Fe Co., New Mexico), and it is the only specimen from its locality in the AMNH collections.

**Fig 4:**
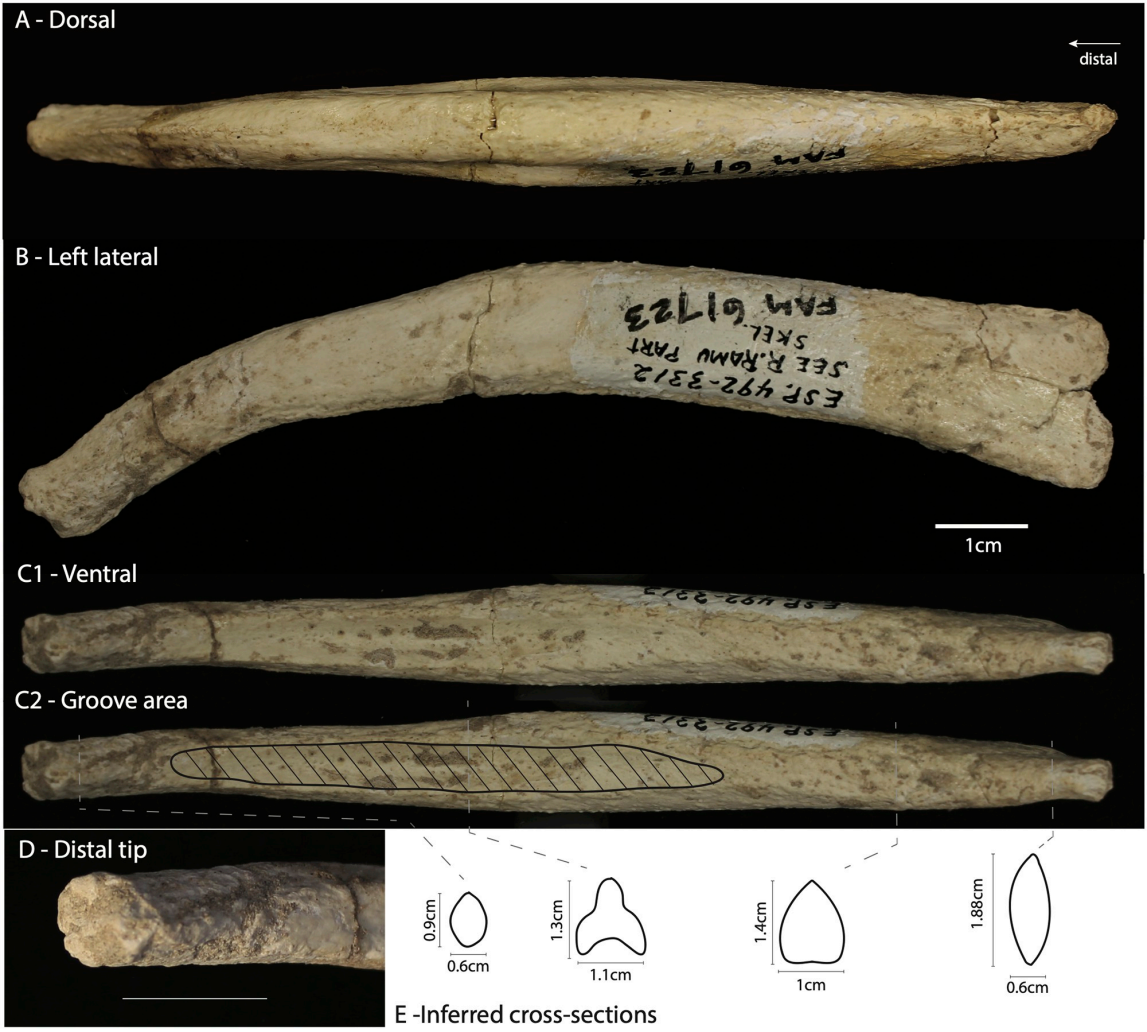
Aelurodon ferox. The baculum of specimen F:AM 61723. A) Dorsal view showing the lateral expansion on the middle part of the shaft where the ventral groove is present. B) Lateral view showing the ventral curvature and the tapering of the bone distally. C) Ventral view showing the urethral groove. Same scale applies to A,B and C. D) Closer view of the distal tip (1cm scale bar). E) Inferred cross-sections at four points along the bone with caliper measurements indicated.

While Wang *et al.* [19] mentioned the presence of a “partial baculum”, the specimen appears to be mostly complete, missing only a minimal portion of the proximal end. It is a robust bone measuring 11.7 cm in length. When observed in lateral view, it curves ventrally and tapers distally (Fig 4). Soft tissue attachment scars are not visible at the proximal end, although the bone seems slightly rougher at that end. A urethral groove is not visible at the proximal end, but begins about 2cm from the proximal end and becomes deeper until about two thirds along the shaft, where it again becomes shallow until it disappears where the distal curvature becomes strongest. Where the groove is present, there is a lateral expansion of the lower (ventral) half of the bone which leaves the dorsal crest visible. The dorsal (and ventral) view reveals that the proximal end is laterally compressed where the groove is absent. The distal end is almost cylindrical in cross section. The distal face of the distal end shows rougher bone, similar to some modern canids.

### Aelurodon stirtoni

**Fig 5:**
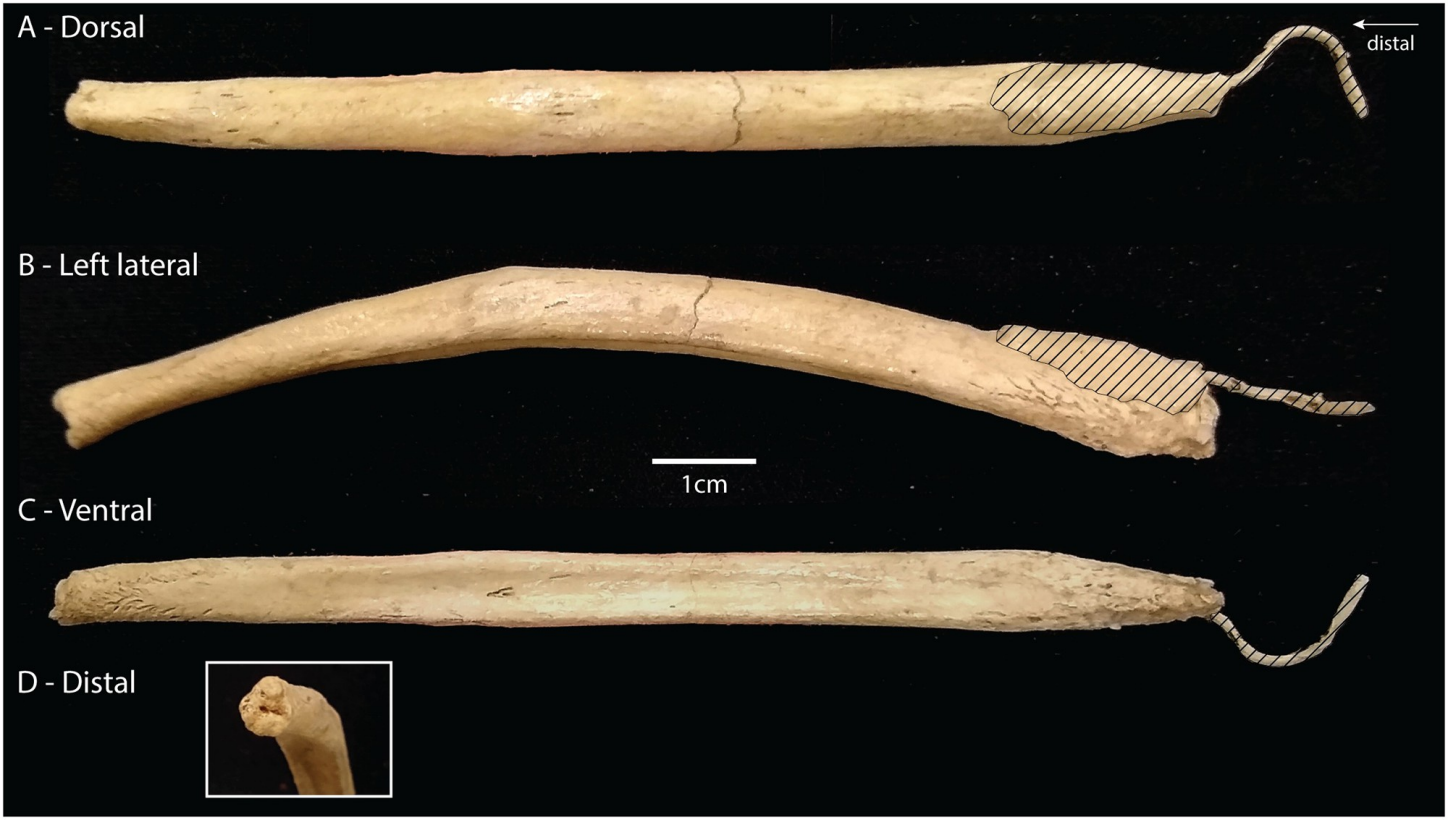
Aelurodon stirtoni. The baculum of specimen USNM PAL 215320. Dashed area indicates material added to the specimen so that it could be mounted as part of an exhibit. A) Dorsal view, which indicates the lack of a lateral expansion seen in other species where the urethral groove is present. The mount obscures details of the proximal end. B) Left lateral view showing the ventrally curved profile. C) Ventral view showing the presence of a shallow groove along most of the length of the shaft but that disappears at both the distal and proximal ends. D) View of the distal end. There was insufficient information to infer cross-section shapes. Images courtesy of the Smithsonian Institution.

*Aelurodon stirtoni* was also a hypercarnivorous species that likely crushed bones when feeding and that could take prey larger than themselves potentially as social hunters, but was smaller than *A. ferox* with approximately 20kg [22]. The baculum of specimen USNM PAL 215320 (Fig 5) was found with an almost complete skeleton, with good preservation quality, including a partial skull and mandible with all teeth preserved [19], as well as stomach content. It is from the late late Barstovian (15.97-13.6Ma, middle Miocene) and was found in the Burge Member of the Valentine Formation, Cherry Co., Nebraska [19]. Although several parts of the skeleton have already been described and imaged [30], the baculum has not. My description of the baculum is based on pictures provided by the Smithsonian National Museum of Natural History. The specimen is on display at that museum and the mount added to position the baculum obscures the dorsal side of the proximal end.

The specimen is complete and has a simple shape. In lateral profile it curves ventrally, showing little variation in dorso-ventral height along the shaft. On the ventral side a shallow groove is present along most of the shaft, disappearing just before the proximal and distal ends. In contrast to the bacula described above, the baculum in this species lacks the lateral expansions typically associated with the urethral groove, and thus there is no dorsal crest. The proximal end is triangular and displays rougher bone that is typically associated with soft tissue attachment. The distal end has an approximately circular circumference (although slightly pinched on the dorsal side) and consists of rough and porous bone.

### Carpocyon compressus

**Fig 6:**
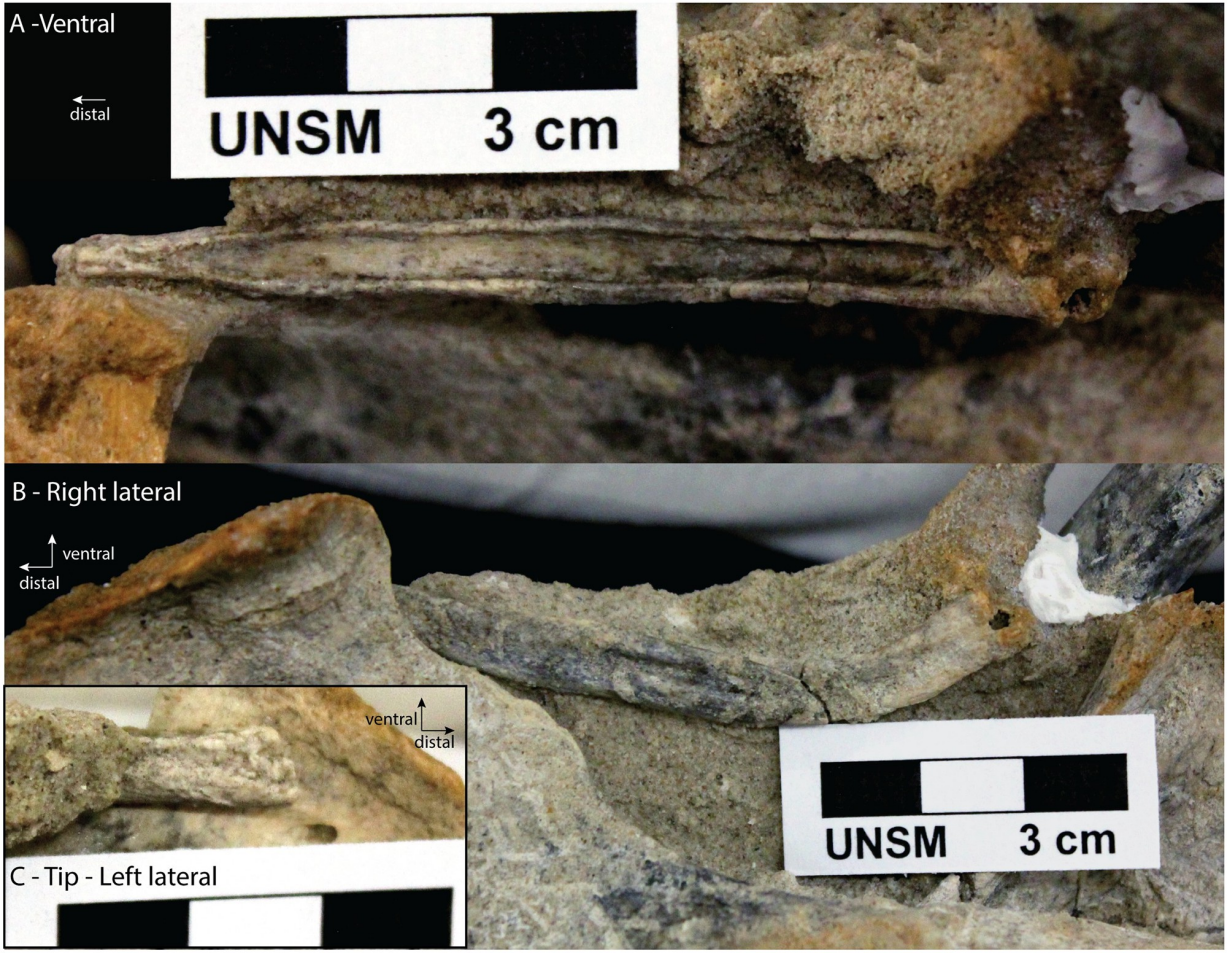
Carpocyon compressus. The baculum of specimen UNSM 128864. A) Ventral view showing a deep urethral groove. B) Right lateral view, with the ventral side facing up on the picture, showing the baculum’s ventral curvature. C) Distal end in left lateral view. There was insufficient information to infer cross-section shapes.

*Carpocyon compressus* was a medium sized species [about 16kg, 22] with dental adaptations indicating a reversal to a hypocarnivorous diet [19]. The baculum of one specimen (UNSM 128864, Fig 6) was found with an almost complete and associated skeleton, between the hind limbs and near the pelvic bones. The specimen also has a complete skull and mandible that allowed its identification [19, where they refer to the specimen field number UNSM 2556-90]. It is late Barstovian (15.97-13.6Ma, middle Miocene), from the Valentine Formation, Brown County, Nebraska [19]. Here I describe the baculum based on photographs provided by the University of Nebraska State Museum. The baculum is partially embedded in matrix along with other post-cranial bones.

The specimen appears to be complete but has one break close to the proximal end. This break slightly alters the lateral profile, but a ventral curvature is clear. The ventral side contains a deep urethral groove that extends for almost the entire length of the bone, ending about 1 cm before the distal end and about 1 cm from the proximal end. Although the dorsal side is still embedded in matrix, in lateral view lateral expansions associated with the groove can be seen, as can the corresponding dorsal crest. The matrix covering the proximal end makes it difficult to determine if there were any soft tissue attachment scars. The distal tip is laterally compressed but has a height similar to the height along most of the shaft. This is in contrast to most canid bacula, which tend to show compressed and cylindrical distal ends.

## Discussion

**Fig 7:**
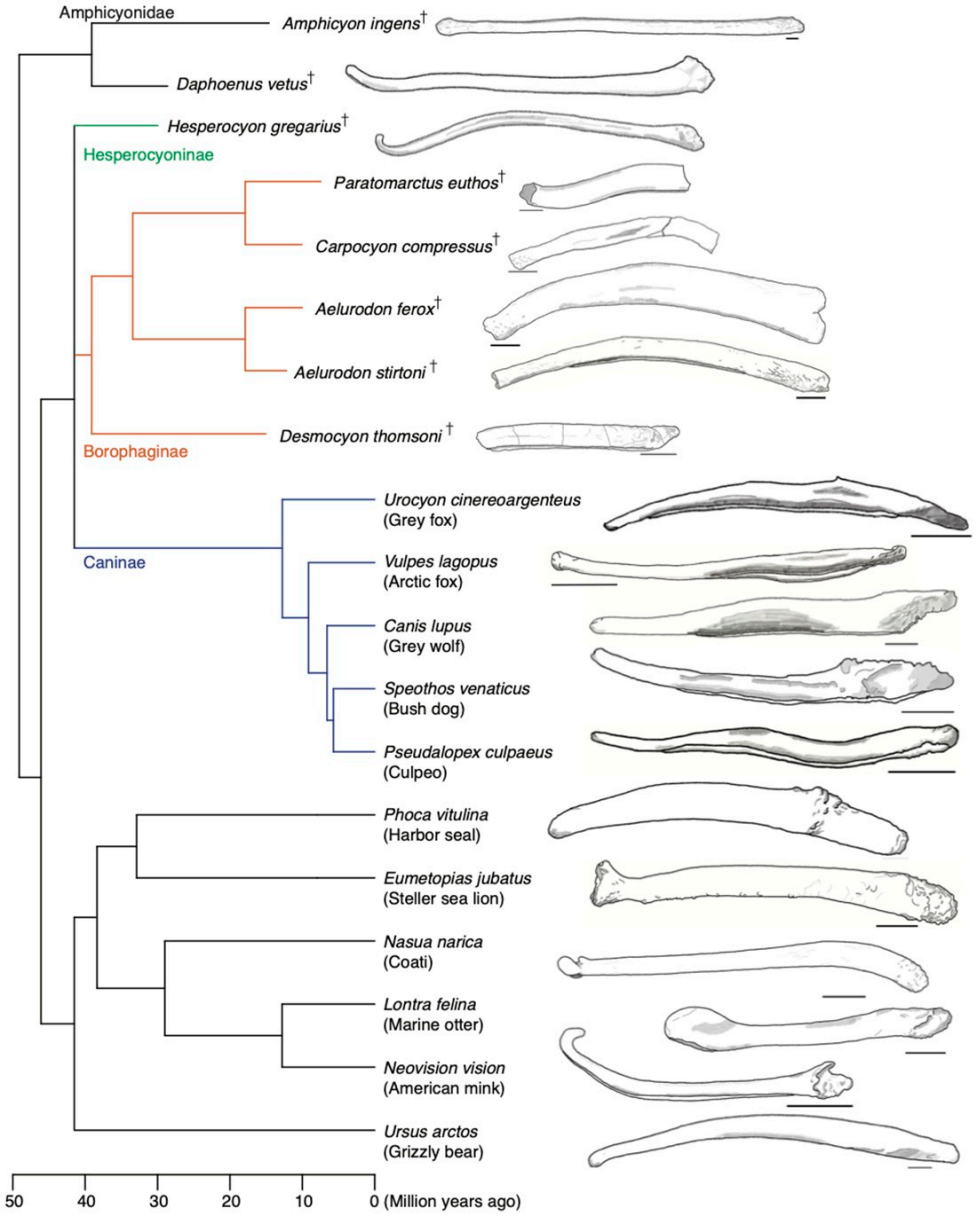
Lateral view silhouettes of bacula of borophagines and selected species within the suborder Caniformia. The specimens represented were chosen to illustrate a substantial range of morphological variation of the canid bacula as well as a few representative morphologies observed in other families. [For further examples of morphological variation of bacula refer to 24, 26, 31]. Drawings for the Borophaginae are based on photographs in this paper. Silhouettes from extant species are based on specimens observed at the Museum of Vertebrate Zoology, Berkeley, California. Drawings from extinct species are based on published images for *Amphicyon ingens* [14], *Daphoenus vetus* and *Hesperocyon gregarius* [32]. The underlying phylogeny is for illustrative purposes and it is based on: Slater [21] for the relationship between the Borophaginae, Caninae and Hesperocyoninae; Slater and Friscia [33] for the position of Ursidae (grizzly bear), Mustelidae (marine otter and american mink), and Procyonidae (coati); Paterson *et al.* [34] for position of the Pinnipedia; and Tomiya and Tseng [35] for the position of the extinct Amphicyonidae (*Amphicyon* and *Daphoenus*). Time scale in million years ago, with ranges of extinct species projected to last fossil occurrences of the taxon.

The bacula of the five borophagine species described here display similarities to each other and to the bacula of all other canids. Specifically, the bacula of extant canines, the extinct hesperocyonine *H. gregarius*, and borophagines are similar in terms of: *i*) relatively large size; and, *ii*) a ventral urethral groove along much of the length of the shaft but not at the distal tip. In two traits the five borophagine bacula are similar to *H. gregarius* but differ from the extant canines: *i*) their ventral curvature (concave down) in lateral profile; and *ii*) the closed urethral groove at the proximal end. Finally, the simple and mostly straight distal end of borophagine bacula is similar to extant canids but differs from the up curved “hook like” distal end of *H. gregarius*’ baculum [16]. Fig 7 shows lateral view silhouettes of these bacula for comparative purposes, with example bacula of species in other families included to capture some of the variation of bacular morphology. Below I discuss how morphological features of borophagine bacula may have implications for their reproductive biology.

### Relative size

Similar to modern canids, the bacula of the Borophaginae were large and robust bones. This supports the observation from Wyss and Flynn [36] that a large baculum is generally diagnostic for the Suborder Caniformia (which includes Canidae, Ursidae, Musteloidea, Pinnipedia, and Amphicyonidae) and contrast with the generally reduced bacula of the sister group, the Feliformia.

#### Implications for reproductive biology

Several studies have explored the relationship between baculum length and the reproductive biology of the living carnivorans [*e.g.*, 8–10, 37–40]. These relationships can thus be used to draw inferences about the reproductive biology of the Borophaginaes. From a functional standpoint, the baculum adds rigidity to the erect penis, therefore the longer and more robust it is, the more it can aid in the maintenance of long-lasting copulations. For example, in extant carnivoran species with long-lasting copulations (defined as more than 3 minutes) tend to have longer bacula [whether phylogeny is considered 40, or not 37], but this relation-ship is not significant when using duration of copulation as a continuous character [9, 38].

Among extant carnivorans, there is a weak correlation between longer bacula and larger testis mass, when both body mass and phylogeny are accounted for [10, 39, but see 8, who found just a weak signal for this relationship]. Larger testis mass is frequently interpreted as a good indicator of the strength of sperm competition because increased sperm production increases the chances of fertilization when females may mate with more than one male during a single round of reproduction [reviewed in 11, 41].

Both of these relationships—copulatory duration and likelihood of sperm competition—suggest that the baculum plays a role in postcopulatory sexual selection, especially in species in which longer copulations may function as a form of mate-guarding that prevents a female from mating with additional males [42] and/or greater sperm production to increase the odds of paternity over competitors. Given that these mechanisms of postcopulatory sexual selection are correlated with large bacula, the relatively large size of bacula within the Borophaginae suggests that similar mechanisms may have been present in these extinct taxa. Given the limited sample size for the Borophaginae and the relatively weak relationships between baculum length and these aspects of postcopulatory competition in extant carnivorans [8], I have refrained from using these relationships to estimate actual values for copulatory duration and testis mass for the Borophaginae.

### Urethral groove

The urethral groove is present in all bacula described from canids, including *H. gregarius* [16], the borophagine specimens examined here, as well as the extant and extinct canines [24–26]. This suggests that this trait is plesiomorphic for the family.

Interestingly, a groove is also present in other families within the Suborder Caniformia, including the extinct and basal Amphicyonidae [13, 14], some mustelids, and pinnipeds. Canids, however, appear to have a unique configuration of the urethral groove. Based on published descriptions [*e.g.* 24, 26] and my own observations of the bacula of the other caniform families, the canids appear to be the only taxon in which the urethral groove is deep, runs for most of the length of the baculum, and is absent at the distal end. While the configuration of the canid urethral groove appears to be unique, it does show variation, mostly in its depth, width, and whether it terminates before the proximal end or not. For example the groove of *Aelurodon* is shallower than in other canids.

In contrast, in the members of other carnivoran families that have a deep groove, the groove continues to the distal most tip of the baculum, and may even be restricted to the distal tip, for example, extant otters (*Lontra*, *Lutra*, and *Pteronuria*), weasels (*Mustela*), and bearded seals (*Erignathus barbatus*) (personal observation). The deep distal groove is also observed in two fossil bacula described from the Amphicyonidae [*Amphicyon ingens and Daphoenus*, 13, 14, respectively], a stem family of the Caniformia [35]. Finally, some bears (Family Ursidae—*e.g.*, spectacled bear *Tremarctos ornatus*, and polar bear *Ursus maritimus*, personal observation) have a urethral groove in a similar position (on the middle of the shaft, absent at the distal end), but much shallower than that of canids.

Finally, while most canine species have an open urethral groove at the proximal end [*e.g.* 26], there are two exceptions, the gray fox [*Urocyon cinereoargenteus*—26] and the bat-eared fox (*Otocyon megalotis*—personal observation), both of which are more similar to the borophagine condition of having a closed proximal end. Interestingly, the gray fox is the most basal species in phylogenetic reconstructions of canines that include fossils [*e.g.*, 43], but see [44], for a discussion of the position of the root of extant canids phylogeny], suggesting that the transition from a closed to an open proximal end might have occurred within the Caninae.

#### Implications for reproductive biology

It has been suggested that the urethral groove protects the urethra during copulation, facilitating the delivery of sperm [37]. Functional experiments support this inferred function in some bats [45], although the shape and position of the groove in these species is different from that of canids. *In silico* functional models of grooves more similar to those seen in canids [9] have demonstrated that the groove increases the robustness of bacula subject to dorso-ventral forces. Given this result, and given that robustness is correlated with long-lasting copulations [9], it seems likely that a reinforced baculum with a urethral groove that aided in the delivery of sperm and enabled prolonged copulation was ancestral to the divergence of the three subfamilies of canids.

The presence of a urethral groove may also have implications for borophagine mating systems. The urethral groove is a major contributor of bacula complexity when its morphology is quantified from 3D digital scans [8, 46]. Brassey *et al.* [8] found that socially monogamous species tend to have bacula that are slightly more complex than solitary species, while species that live in groups with several breeding females tend to have bacula less complex than the other two mating systems. If this relationship also applies to the borophagines then their deep urethral groove would suggest that they did not have a mating system with multiple breeding females, although this inference remains tentative.

### Distal end

The distal end of the borophagine baculum shows a simple morphology, either mostly cylindrical or laterally compressed, which is similar to the simple distal end observed in extant canids [26]. In contrast, the extinct hesperocyonine *H. gregarius* had a dorsally-curved distal tip (Fig 7, [16, 32]), which is much more complex than other known canid bacula and more similar to that of some extant mustelids and procyonids.

#### Implications for reproductive biology

The distal end of the baculum of extant carnivorans is associated with the mode of ovulation. While species with more complex distal tips tend to show induced ovulation, those with simple distal tips are associated with spontaneous ovulation [8]. Given that most extant canids have spontaneous ovulation, the bacula described here suggest a similar strategy for the borophagines. Larivière and Ferguson [47] hypothesized that induced ovulation is advantageous for species with low population densities and with a multi-male mating system that live in highly seasonal environments [see also 48]. Although their hypothesis does not entail that high population densities are associated with spontaneous ovulation, species with higher population density have higher chances of mate encounters during the restricted time window of spontaneous ovulation. The fossil record of borophagines is particularly rich [22], consistent with the potential for high population densities in these animals. Together with the bacular structure of these animals, available evidence, although limited, suggests that the borophagines were spontaneous ovulators.

### Curvature

All bacula described here have a primarily ventral curvature (arched) in lateral profile (Fig 7), with one species (*P. euthos*) having a secondary dorsal curvature on the distal end. Only two specimens (both *Aelurodon*) have complete bacula without any indication of preservational distortion (such as bending or warping), while for the other three species the bacula are either incomplete or had the lateral profile slightly altered by a break (*C. compressus*). Nevertheless, a primarily ventral curvature is seen in all specimens. In this regard, the bacula of the Borophaginae differ from the mostly straight or even dorsally curved bacula of almost all extant canids (Fig 7). For the very few extant canids (such as the gray fox) with a ventrally curved baculum, it is not as curved as in *Aelurodon*. The degree of ventral curvature in the borophagines is present in some bears and pinnipeds, although more data are necessary to establish the generality of this observation.

#### Implications for reproductive biology

Using *in silico* biomechanical analyses, Brassey *et al.* [9] showed that a ventral curvature leads to less robust bacula when a dorso-ventral force is applied to its distal tip. In turn, species with less robust bacula show shorter duration of copulation [9]. Based on this feature alone, it is possible to hypothesize that the baculum of borophagines would not withstand as much force during copulation as modern canids and thus had a shorter duration of copulation than extant canids. However, other morphological features may have increased the robustness of borophagine baculum, which would suggest a long copulatory duration. For example, the baculum of *Aelurodon ferox* (Fig 4) is a massive bone, with a thick dorsal ridge and a dorso-ventral height that is larger than observed in modern carnivorans. Further quantitative analysis similar to those of Brassey *et al.* [9] should help clarify the robustness of borophagine bacula, and thus inferences about their copulatory duration.

### Conclusion

The baculum of borophagines shows an overall similarity with extant canines, in relative size, a simple distal end, and presence and configuration of a urethral groove. Drawing upon correlations between bacular morphology and reproductive biology of the extant carnivorans, it is possible to generate preliminary inferences regarding some aspects of borophagine reproductive biology. Specifically, based on comparative bacular morphology, I suggest the borophagines likely had long copulatory durations and spontaneous ovulation, based on the size and simple distal tip of their bacula, respectively. It is possible that with the description of more bacula and further quantitative analysis more robust inferences can be made. The study of reproductive biology in extinct species is challenging and depends on well established relationships between morphology and behaviour or physiology. Nevertheless, further inferences for extinct species could improve our understanding of the selective pressures and consequent evolutionary dynamics of reproductive strategies changing through time. In the long term, it might be possible to better distinguish the role of reproductive function from “phylogenetic inertia” in determining the morphology of the baculum.
